# Role of CD248 as a potential severity marker in idiopathic pulmonary fibrosis

**DOI:** 10.1186/s12890-016-0211-7

**Published:** 2016-04-14

**Authors:** Domokos Bartis, Louise E. Crowley, Vijay K. D’Souza, Lee Borthwick, Andrew J. Fisher, Adam P. Croft, Judit E. Pongrácz, Richard Thompson, Gerald Langman, Christopher D. Buckley, David R. Thickett

**Affiliations:** Respiratory Research Group, Centre for Translational Inflammation and Fibrosis Research, University of Birmingham, Birmingham, United Kingdom; Department of Pharmacological Biotechnology, Szentágothai Research Centre, University of Pécs, 20 Ifjusag Utja, H-7624 Pécs, Hungary; Fibrosis research group, Institute of Cellular Medicine, Newcastle University, Newcastle upon Tyne, United Kingdom; Institute of Transplantation, Newcastle Upon Tyne Hospitals NHS Foundation Trust, Newcastle upon Tyne, United Kingdom; Rheumatology Research Group, Centre for Translational Inflammation and Fibrosis Research, University of Birmingham, Birmingham, United Kingdom; Department of Heart & Lung Transplantation, University Hospital Foundation NHS trust Birmingham, Birmingham, United Kingdom; Department of Pathology, Heart of England foundation NHS trust, Birmingham, United Kingdom

**Keywords:** IPF, Idiopathic pulmonary fibrosis, TGF-beta, CD248, Endosialin, Biomarker

## Abstract

**Background:**

CD248 or Endosialin is a transmembrane molecule expressed in stromal cells binding to extracellular matrix (ECM) components. It has been previously implicated in kidney fibrosis, rheumatoid arthritis as well as in tumour-stromal interactions. This study investigates the role of CD248 in the pathogenesis of fibrotic diseases in Idiopathic Pulmonary Fibrosis (IPF).

**Methods:**

CD248 quantitative immunohistochemistry (IHC) was performed on lung samples from 22 IPF patients and its expression was assayed in cultured pulmonary fibroblasts and epithelial cells. Effects of CD248 silencing was evaluated on fibroblast proliferation and myofibroblast differentiation.

**Results:**

IHC revealed strong CD248 expression in mesenchymal cells of normal lung structures such as pleura and adventitia but not in epithelium. Fibrotic areas showed markedly stronger staining than unaffected lung tissue. The extent of CD248 staining showed a significant negative correlation to lung function parameters FEV1, FVC, TLC, and TLCO (r2 > 0 · 35, *p <* 0 · 01). CD248 protein levels were significantly greater in IPF-derived lung fibroblasts vs normal lung fibroblasts (*p <* 0 · 01) and CD248 silencing significantly reduced the proliferation of lung fibroblasts, but did not affected myofibroblast differentiation.

**Conclusion:**

We conclude that CD248 overexpression is possibly involved in the pathogenesis of IPF and it has potential as a disease severity marker. Given that CD248 ligands are collagen type I, IV and fibronectin, we hypothesise that CD248 signalling represents a novel matrix-fibroblast interaction that may be a potential therapeutic target in IPF.

**Electronic supplementary material:**

The online version of this article (doi:10.1186/s12890-016-0211-7) contains supplementary material, which is available to authorized users.

## Background

Idiopathic pulmonary fibrosis (IPF) is the most common form of interstitial lung diseases (ILD) with a worldwide incidence ranging between 14–43 per 100 000 individuals and an average incidence of 7.44 per 100 000 in the UK [1]. A recent clinical data analysis of UK patient cohorts by Navaratnam *et al.* showed a yearly 5 % increase in incidence which was independent of the ageing of the population [2].

Lung function progressively deteriorates in IPF patients leading to respiratory failure and finally death with a median survival around three years from diagnosis [3]. Therapeutic options for IPF are limited to date, with pirfenidone and nintedanib being the only medications which seem to slow down disease progression and they have only been tested in patients with mild disease [4, 5]. These drugs are both expensive and real life usage suggests that toxicity leading to drug discontinuation is higher than reported in the licensing trials [6].

The pathophysiology of IPF is complex involving fibroproliferation, epithelial cell apoptosis, epithelial-mesenchymal transition (EMT), neo-angiogenesis, and intra-alveolar coagulopathy [7–9]. In fibrotic tissue, effector cells are activated fibroblasts or myofibroblasts residing in large numbers in fibroblastic foci, a characteristic feature of usual interstitial pneumonia. These alpha smooth muscle actin (α-SMA)-expressing cells secrete large amounts of collagen and other extracellular matrix (ECM) components and are thus considered to be responsible for pathological tissue remodelling [10]. Pro-fibrotic growth factors including transforming growth factor-beta 1 (TGF-β1) and platelet-derived growth factor (PDGF) are believed to drive the fibro-proliferative process in IPF and promote fibroblast-to-myofibroblast differentiation and collagen deposition [9, 10]. The degree of involvement of EMT in IPF pathogenesis—particularly the number of fibroblasts and myofibroblasts derived from transdifferentiated epithelial cells—is currently debated [7, 11, 12].

CD248 (also termed Endosialin and TEM-1), is a heavily glycosylated transmembrane protein that was identified initially as a cell surface marker overexpressed in tumour vasculature [13]. Later it was revealed that in fact CD248 was expressed by pericytes, and not the underlying endothelium [14]. CD248 is now regarded as a mesenchymal marker, expressed on various fibroblast types, pericytes, mesenchymal stem cells, smooth muscle cells and osteoblasts [15, 16]. CD248 is also implicated in the regulation of the proliferation of mesenchymal elements [17, 18].

CD248 was demonstrated to be overexpressed in various mesenchymal cells in pathological conditions including sarcomas [19], inflammation [20] and fibrosis [21]. In renal fibrosis CD248 expression was found to relate to the severity of the disease [21]. There is no information about CD248 expression in lung disease especially IPF. We hypothesised that CD248 expression would be a marker of severity in IPF. We also investigated its role in fibroproliferation and EMT.

## Methods

### Patient samples

Lung tissue samples for histology were obtained by diagnostic video assisted thoracoscopic (VATS) lung biopsy and transplant explants. These procedures were performed by surgical teams at Heartlands Hospital and the Queen Elizabeth Hospital, Birmingham between the time-frame January 2005 to December 2012. Lung resections were fixed in formalin, embedded into paraffin and sectioned according to standard procedures. A total of 22 patients’ samples were included in the study. Demographic and clinical data of patients were obtained from electronic and paper records. Patient data is summarized in Table 1. No patients received disease modifying treatment (pirfenidone or nintedanib) prior to surgery.

**Table 1:** Patient demographics and clinical data. We analysed the demographic and clinical data of 22 patients with a histologically confirmed IPF diagnosis. 11 of the patients underwent VATS and had mild-to-moderate fibrotic changes in the lung. Another 11 IPF patients underwent lung transplantation because of severe or end-stage fibrosis. There was no significant difference between the patient groups’ age, smoking status and body mass index (BMI). The lung function parameters Forced Expiratory Volume in 1 s (FEV1), Forced Vital Capacity (FVC), Total Lung Capacity (TLC) and Transfer factor for carbon monoxide in the lung (T_LCO_) were significantly higher in the patient group with mild-to-moderate fibrotic changes compared to those with severe or end-stage fibrosis (*p <* 0.01 using Students unequal variance *t*-test)

	Patients with mild-to-moderate fibrosis (VATS biopsy patients)	Patients with severe or end-stage fibrosis (Lung transplant patients)	p-value (Students *t*-test)
Demographic and clinical data			
N	11	11	-
Sex	4 males, 7 females	9 males, 2 females	-
Median age (range)	66 (39–76)	58 (44–61)	0.126
Smoking in history (percent)	7 (63 %)	7 (63 %)	0.482
Pack years (mean ± SEM)	28.37 ± 11.97	21.93 ± 6.61	0.784
BMI (mean ± SEM)	28.87 ± 1.37	27.67 ± 1.87	0.636
Lung function			
FEV1 (mean ± SEM)	2.51 ± 0.19	1.59 ± 0.20	0.0009
FVC (mean ± SEM)	3.21 ± 0.32	1.92 ± 0.23	0.0006
TLC (mean ± SEM)	4.77 ± 0.54	3.04 ± 0.34	0.0014
TL_CO_ (mean ± SEM)	3.69 ± 0.36	2.09 ± 0.30	0.0099
Medication (given only after biopsy)			
Prednisolone	6 (54.5 %)	8 (72.7 %)	-
Azathioprine	3 (27.2 %)	4 (36.3 %)	-
N-acetylcysteine	4 (36.3 %)	2 (18.1 %)	-

Primary human lung fibroblasts were isolated from explanted lung tissue of patients with IPF, acquired at the time of lung transplantation (*n =* 6) and lung tissue from healthy donors rejected for transplantation as controls (*n =* 6) at the Institute of Transplantation, Newcastle Upon Tyne Hospitals NHS Trust. All procedures in this study were performed in accordance with approval from the local research ethics committees at the University of Birmingham and Newcastle University, respectively. All patients gave written informed consent for the use of their tissue and clinical data for research purposes. Ethics committee approval number is 07/MRE08/42.

### Immunohistochemistry and digital image analysis

Lung tissue samples for immunohistochemistry (IHC) were obtained by VATS from patients with mild-to-moderate fibrosis (*n =* 11) or were taken from lungs of patients with end-stage fibrosis undergoing lung transplantation (n = 11). Sections were processed for CD248 immunohistochemical staining using standard methods for diaminobenzidine (DAB) chromogen and haematoxylin for nuclear staining. The B1/35.1 anti-human CD248 antibody [14] was used for IHC in an automated IHC staining system (Roche Benchmark Ultra). The area staining positive for CD248 was compared between the two patient groups using the ImageJ software for digital image analysis as follows: we took 3–7 high resolution digital images per slide at random locations. Patient identification were blinded during the whole analysis. CD248 signal and haematoxylin signal on the images were digitally separated using the Colour Deconvolution plugin for ImageJ [22]. Threshold value was set manually at the same levels in both of the channels on all of the images. The area above the threshold was summarised for each image, representing the area stained positive for CD248 or haematoxylin on each image. CD248 positive area was compared to the haematoxylin staining area representing relative CD248 expression in the lung samples of IPF patients. A graphical explanation of this method is shown on Fig. 2a. The relative CD248 staining area were compared between transplant and VATS biopsy patients. We used both Student’s *t*-test and Mann–Whitney *U*-test for statistical analysis of the data derived from images, where *p <* 0.05 denoted statistical significance.

### Cell culturing and treatments

Normal human lung fibroblasts (NHLF) were purchased from Promocell (Heidelberg, Germany). At least 3 batches from different patents were used for every experiment. NHLFs were initially cultured and expanded in Fibroblast Growth Medium (Promocell) according to the supplier’s instructions. A549 human lung adenocarcinoma cell line was maintained in DMEM supplemented with 2 mM L-glutamine, HEPES, non-essential amino acids, 100 U/ml penicillin and 100 mg/ml streptomycin and 10 % FCS.

Additional primary fibroblasts were isolated at Newcastle University from the lungs of patients with advanced IPF and the lung tissue from healthy donors rejected for transplantation as controls at the Institute of Transplantation, Newcastle Upon Tyne Hospitals NHS Trust. The isolation method based on a cell outgrowth technique. Briefly, lung tissue pieces (<1 mm^3^) were cultured in DMEM/F12 (Sigma) supplemented with 10 % FCS, 1 % L-glutamine, 100U/ml penicillin and 100 μg/ml streptomycin to allow cells to migrate out of the tissue. After 7 days the tissue is removed and the cells grown to confluence. Mesenchymal phenotype was confirmed by positive expression of fibronectin, vimentin and α-smooth muscle actin and little/no expression of E-cadherin, ZO-1 and CD45. Fibroblasts were used at the third passage in all experiments.

Human Primary Lung epithelial cells were isolated from tissue samples from lobectomy patients with normal lung function. Cells were isolated and cultured as described elsewhere [23]. TGF-β1 was obtained from R&D Systems (Abingdon, UK). Final concentration of TGF-β1 was 10 ng/ml.

### siRNA transfection

CD248 knock-down (KD) in NHLFs was carried out using specific siRNA (Life Technologies). We used Lipofectamine 2000 reagent and Opti-MEM (both from Life Technologies) according to the manufacturer’s instructions. Cells were cultured after transfection for 48 h. Efficiency of KD was determined using PCR and flow cytometry (Additional file 1: Figure S4A and B, respectively). Cell viability was >95 % after 48 h of siRNA transfection as determined by the trypan-blue exclusion test.

### Proliferation assay

A commercial ELISA-based bromodeoxyuridine (BrdU) proliferation assay kit was used from Calbiochem (Watford, UK) according to the manufacturer’s instructions. Briefly, previously serum-starved cells were seeded into a 96-well tissue culture plate in DMEM + 0.1 % FCS; BrdU and stimulants were added afterwards. Cells were incubated for 24 h to incorporate BrdU, then the proliferation was stopped using the fixative agent supplemented with the kit. The development and colorimetric measurements were performed according to the manufacturer’s instructions. Proliferation experiments were repeated for at least 3 times with cells from 3 different donors.

### Flow cytometry

Cultured cells were detached using a non-enzymatic cell dissociation solution (Sigma Aldrich) to preserve the trypsin-sensitive CD248 epitope. Labelling was carried out using an anti-CD248-FITC monoclonal antibody [24]. Samples were analysed on a CyanADP Analyzer flow cytometer and Summit v4.3 software. CD248 protein expression is presented as median fluorescent intensity (MFI).

### Real time qPCR

Total RNA was isolated from cultured cells using the NucleoSpin RNA isolation kit with on-column DNase digestion (Machery-Nagel), cDNA synthesis was performed using a High Capacity RNA-to-cDNA kit (Applied Biosystems) following manufacturer’s protocols. For real-time qPCR experiments, master mixes with or without SYBR Green were used (Roche). The list of primers is available in the online supplement. (Additional file 1: Table S1) PCR experiments were performed on a Light Cycler 480 Instrument (Roche). In the plots reverse ΔCt values versus GAPDH expression are presented; the calculation formula was reverse ΔCt = Ct(GAPDH) - Ct(Target) [25]. The mean Ct values were calculated for 3 or more independent experiments.

### Statistics

Data were analysed on using SPSS for Windows 16.0 (SPSS, Inc., Chicago, IL). Data were tested for normality using Spearman’s chi squared test and analysed by non-equal variance *t* test or Mann–Whitney *U* test. Data are expressed as mean ± SE unless otherwise indicated. Correlations were calculated using least squares linear regression test.

## Results

### CD248 immunohistochemistry staining pattern on fibrotic lung

**Fig. 1:**
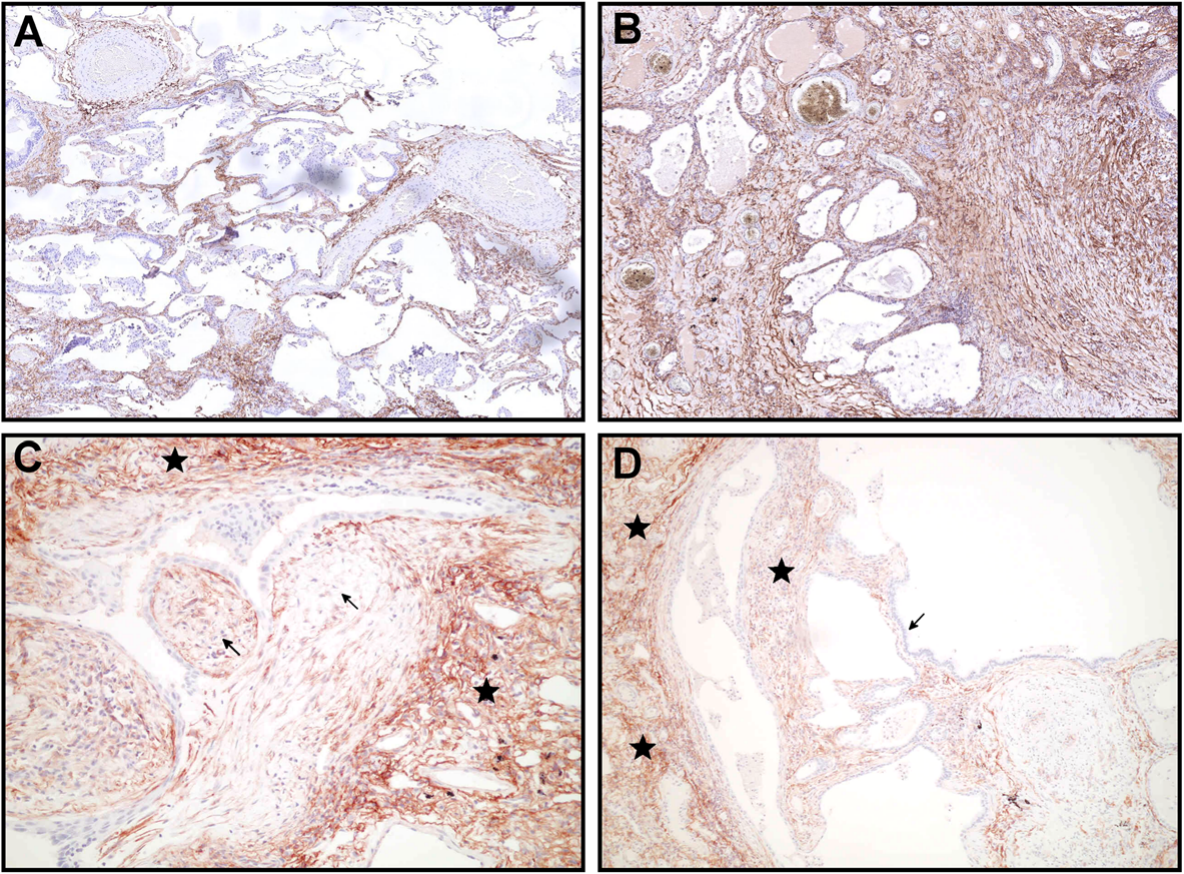
Panel **a**: Typical CD248 staining patterns in the fibrotic lung in a sample obtained by VATS biopsy from an IPF patient with mild/moderate fibrotic changes in the lung. Original magnification 40x.: Panel **b**: Typical CD248 staining in end-stage fibrosis in the lung sample of an IPF patient undergone lung transplantation. Original magnification 40x. Panel **c**: Pale CD248 staining (brown) in fibroblastic foci (arrows) compared to adjacent scarring (stars), (original magnification x200). Panel **d**: IHC staining of CD248 in IPF patient. The epithelium in dilated air spaces is negative for CD248 (arrow) while adjacent fibrotic stromal regions (stars) show pale to moderate CD248 staining. (original magnification x100). Slides were visualized using an Olympus BX51 microscope (Olympus) and photographed with an Olympus C3030 camera (Olympus)

CD248 expression was compared in lung samples from patients with IPF who underwent either VATS biopsy (mild or moderate fibrotic changes, *n =* 11) or lung transplantation (severe or end-stage fibrotic changes, *n =* 11). CD248 expression was observed specifically on fibroblast-like stromal cells but not on epithelium, endothelium, smooth muscle, alveolar macrophages or inflammatory cells (Fig. 1, Additional file 1: Figure S1). Areas of fibrosis stained positive for CD248. There was a range of weak to strong staining in the fibrotic regions with the more established fibrosis showing greater CD248 expression levels. There was a visible difference between CD248 expression patterns in lung samples with mild or moderate fibrosis and severe fibrosis (Fig. 1a and b, respectively.) Fibroblastic foci generally stained less intensive or not at all for CD248 in comparison to the surrounding fibrosis (see Fig. 1c).

In contrast to fibroblasts, epithelial cells in the lung stained negative for CD248 (Fig. 1d) and some of the physiological structures in the lung proved to be consequently positive for CD248, like the adventitia of larger muscular arteries, interlobular septa and pleural regions (Additional file 1: Figure S1). Staining in these latter structures was universally greater than that observed in fibrotic areas. In addition, staining around the bronchioles was seen but areas of inflamed interstitium exhibited very little CD248 expression. (Additional file 1: Figure S1).

### CD248 expression in the lung of IPF patients is significantly associated with the severity of fibrosis and lung function

**Fig. 2:**
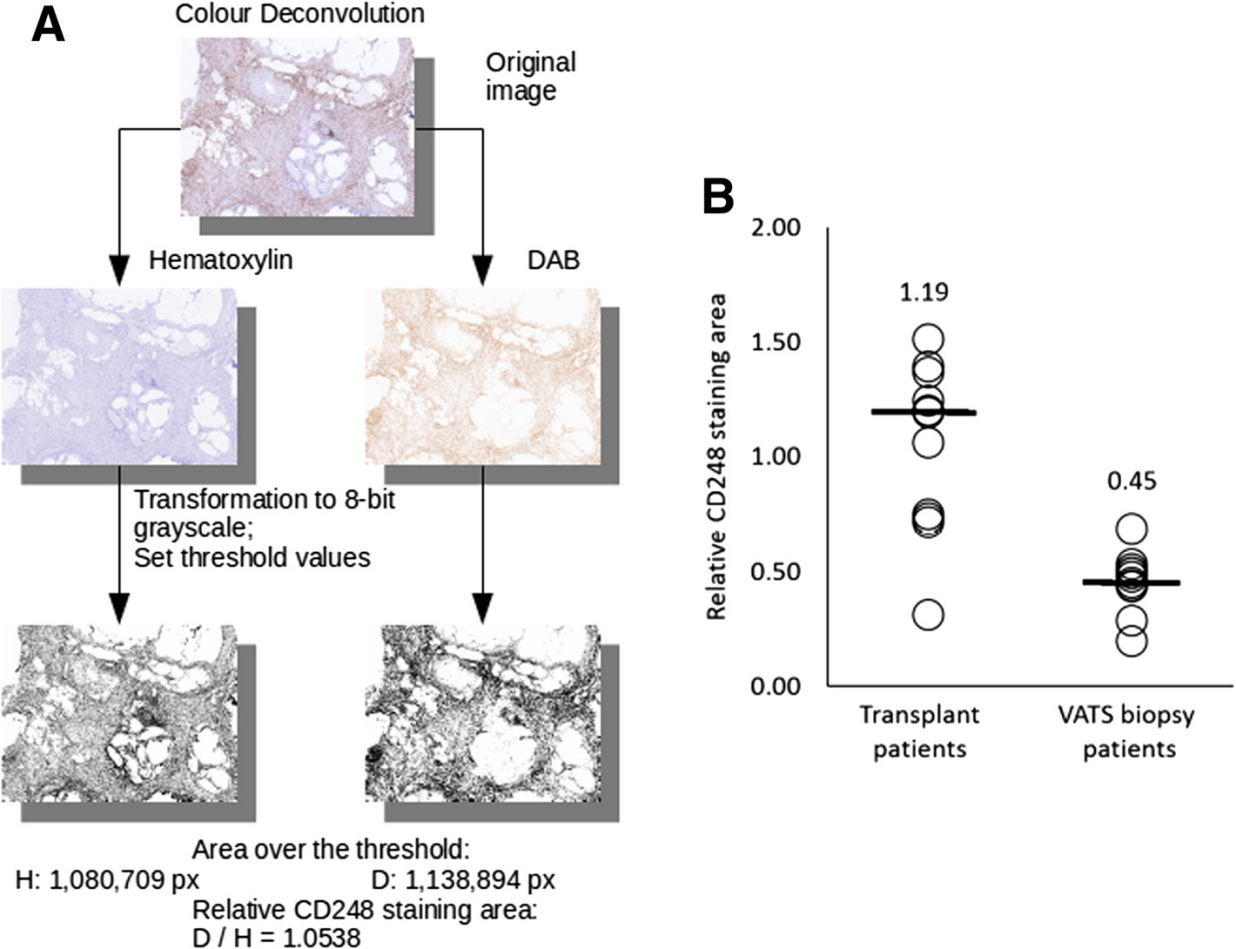
Panel **a**: Graphical explanation of the digital image analysis using the Colour Deconvolution ImageJ plugin. The digital images taken of the IPF sections opened in ImageJ and DAB (brown) and Hematoxylin (blue) channels were digitally separated using the plugin. Then both resulting images were transformed to 8-bit greyscale images and after threshold levels were applied resulting 1 bit images. We compared Hematoxylin-stained and DAB-stained pixel numbers above the threshold on the 2 images so that we get a relative CD248 staining area. Panel **b**: Comparison of the relative areas stained positively for CD248 in IPF patient groups plotted on a dot plot diagram, horizontal lines representing the median value. On the diagram we plotted relative staining areas on sections obtained from IPF patients with mild-to-moderate fibrosis (*n =* 11, samples obtained by VATS biopsy) and lung transplant patients with severe or end-stage fibrotic changes (*n =* 11). In VATS biopsy patients with mild-to-moderate fibrotic changes significantly smaller area (median value = 0.45) stained positively for CD248 when compared to those with severe or end-stage lung fibrosis (median value = 1.19; *p <* 0.05 using Mann–Whitney *U*-test)

Since staining intensity with DAB is not proportional to expression level [22] we compared the relative area stained positive for CD248 by comparing DAB staining (e.g. CD248 positive areas) to the area of nuclear staining with haematoxylin (Fig. 2a). We found, that the area stained positively for CD248 was significantly higher in the transplant explant samples than in patients undergoing VATS biopsy (Fig. 2b) For further statistical information on please see Additional file 1: Figure S2).

Importantly in univariate analysis there was a significant negative correlation with the relative staining area of CD248 with lung function. Our data show that FEV1, FVC, TLCO and TLC are significantly correlated to CD248 staining in fibrotic lungs (r^2^ value between 0.35 and 0.5; *p <* 0.05 using non-parametric ANOVA, detailed data are presented in Table 2 and graphical presentation of the data are in Additional file 1: Figure S3.

**Table 2:** Summary of regression analysis data. The normal distribution of all datasets were analysed and confirmed using the Chi square goodness-of-fit method (data not shown). R squared values were calculated using the least squares linear regression analysis. The significance of the correlation were analysed using ANOVA test

	Correlation to CD248 relative staining area	R squared values (least squares linear regression)	Significance of correlation (ANOVA)
Demographics/clinical data	Age	0.097	0.156
	Smoking (pack-years)	0.084	0.226
Lung function data	TLC	0.432	0.005
	FVC	0.358	0.003
	FEV1	0.392	0.002
	TLCO	0.455	0.008

### CD248 expression levels are higher in fibroblasts derived from IPF patients than those from normal lungs

To confirm that CD248 expression is higher in IPF fibroblasts compared to normal fibroblasts, expression was compared by qPCR and flow cytometry.

**Fig. 3:**
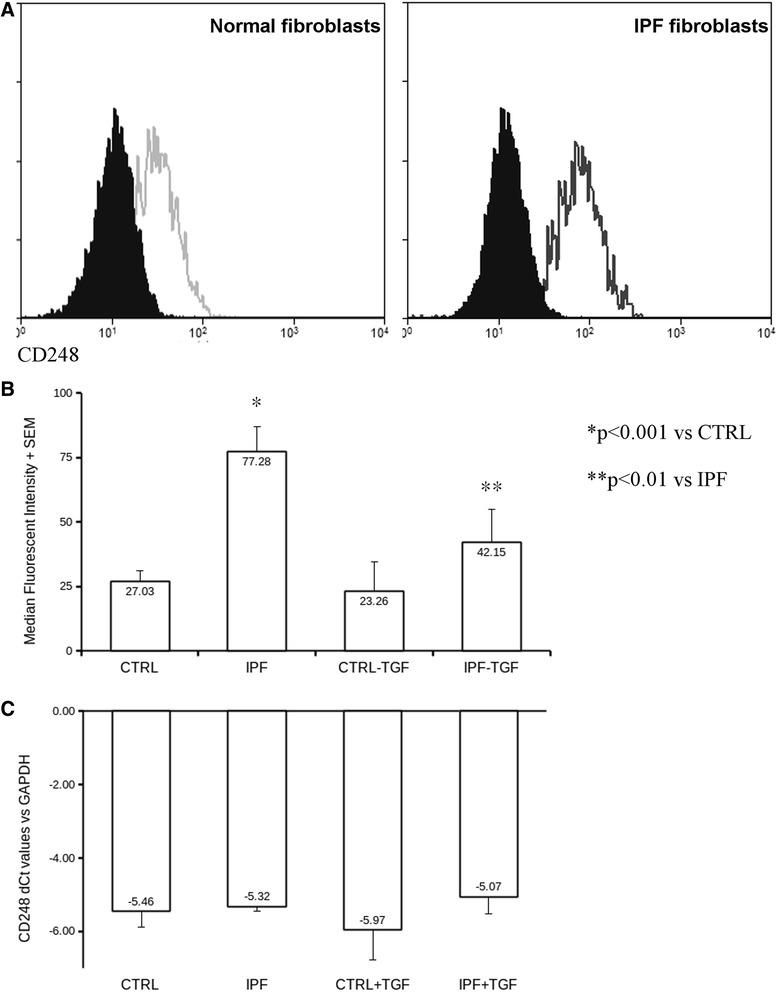
Measurement of expression levels of CD248 on human lung fibroblasts derived from normal (*n =* 6) and IPF lungs (*n =* 6). Panel **a**: Flow cytometric histograms showing CD248 expression on human lung fibroblasts from normal (diagram on the left) and IPF lungs (diagram on the right). Open histograms represent samples labelled with FITC-conjugated CD248 antibody, closed histograms represent FITC-conjugated isotype control antibody. Two representative diagrams are shown of twelve independent samples. Panel **b**: CD248 expression changes in fibroblasts treated with TGF-beta (*n =* 12). Bars represent median fluorescent intensities (average + SEM is shown of six independent samples). CD248 expression levels were significantly higher (*p <* 0.001) in fibroblasts derived from IPF patients than in those from normal lungs. CD248 expression declined significantly (*p <* 0.01) upon TGF-beta treatment in fibroblasts derived from IPF lungs but not in those from normal lungs. Panel **c**: CD248 mRNA expression levels in fibroblasts treated with TGF-β1 derived from normal (*n =* 6) and IPF lungs (*n =* 6). Bars repesent CD248 Ct values compared to GAPDH. Note that there are no significant differences between CD248 mRNA expression in different samples

There was no significant difference in mRNA expression of CD248 between normal and IPF derived fibroblasts (Fig. 3c). In contrast CD248 protein expression was significantly increased when measured using flow cytometry (Fig. 3a and b). Interestingly, CD248 expression seemed to be sensitive to TGF-β1 treatment in IPF fibroblasts but not in normal fibroblasts (Fig. 3b) with TGF-β1 reducing expression.

### CD248 regulates fibroblast proliferation but not myofibroblast transdifferentiation and collagen synthesis in vitro

**Fig. 4:**
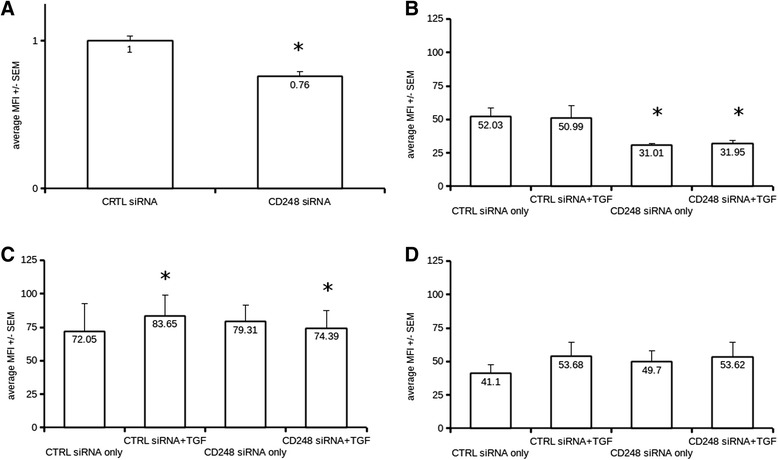
Panel **a**: CD248 knockdown with specific siRNA significantly reduced BrdU uptake in NHLFs (*n =* 3 different NHLF batches, total replicates 18, *p <* 0.001 with Student’s non-equal varienace *t*-test). Cells were incubated with 30 pmol of control or CD248-specific siRNA for 48 h. BrdU was added according to the manufacturer’s recommendation (Calbiochem). BrdU incorporation was detected using an indirect ELISA method. Panel **b**. CD248 expression in NHLF cells measured with flow cytometry (cells from *n =* 5 different donors). CD248-specific siRNA caused significant (*p < 0.05, non-equal variance *t*-test) drop in the protein expression levels when compared to treatment with scrambled control siRNA. Median Fluorescent intensity and Standard error of the mean (SEM) is plotted on the graph. Panel **c**. Intracellular Collagen 1a1 expression levels in NHLF cells measured by flow cytometry (cells from *n =* 5 different donors). TGF-β1 treatment caused a significant elevation in collagen 1 synthesis (*p < 0.01, non-equal variance *t*-test) but we measured no significant difference when cells were treated with CD248-specific siRNA. Median Fluorescent intensity and Standard error of the mean (SEM) is plotted on the graph. Panel **d**. Intracellular alpha smooth muscle (aSMA) expression levels in NHLF cells measured by flow cytometry (cells from *n =* 5 different donors). TGF-β1 treatment caused significant increase in aSMA expression in NHLF cells regardless of siRNA treatment siRNA (*p <* 0.05, non-equal variance *t*-test). Median Fluorescent intensity and Standard error of the mean (SEM) is plotted on the graph

In order to establish a functional role for CD248 in fibroblast function we transfected NHLFs with negative control or CD248-specific siRNA for 48 h, then assayed the bromodeoxyuridine (BrdU) uptake of the cells with ELISA. CD248 expression levels after knockdown were assessed with qPCR and FACS (Additional file 1: Figure S4). We found, that CD248 knockdown significantly reduced NHLF BrdU uptake (Fig. 4a). We tested, whether CD248 knockdown had any effect on in vitro myofibroblast transdifferentiation by assessing alpha smooth muscle actin (alpha-SMA) and collagen-1 mRNA and protein levels. We found, that CD248 knockdown did not induce myofibroblast differentiation as there were no significant differences in collagen and alpha-SMA levels between control and CD248 KD samples.

### CD248 expression is not induced by EMT in lung epithelial cells

**Fig. 5:**
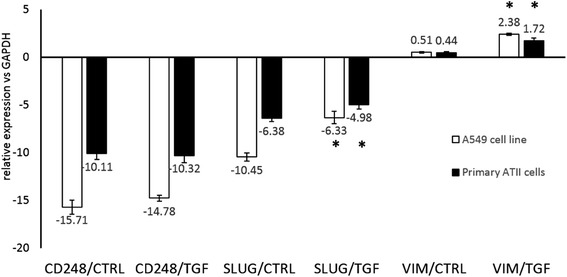
CD248 expression do not changes significantly in A549 (*n =* 3) or in primary type II alveolar epithelial cells (ATII, *n =* 6) upon TGF-β1 – induced EMT. Cells were treated with 10 ng/ml TGF-β1 for 72 h. mRNA expression levels of CD248, SLUG Vimentin (VIM) and GAPDH were tested with reverse transcription quantitative PCR (RT-qPCR). On the graph, reverse dCt values are plotted; values were calculated as described above. CD248 expression levels were very low and did not changed significantly upon growth factor treatments. In contrast, expression levels of EMT markers SLUG and VIM were significantly elevated (*p < 0.05) in the TGF-β1-treated samples, indicating that epithelial cells have undergone EMT

CD248 is a mesenchymal marker expressed on fibroblasts but not on epithelial cells in the lung. We tested whether CD248 is expressed by pulmonary epithelial cells undergoing epithelial-mesenchymal transition in vitro using both reverse-transcription qPCR and flow cytometry. We found that CD248 mRNA is expressed in epithelial cells at a very low level and protein expression is negative with flow cytometry. Addition of TGF-β1 did not induce CD248 expression but induced changes characteristic for EMT (Fig. 5).

## Discussion

In this study we have evaluated CD248 expression in the lungs of patients with mild early stage IPF and lung transplant explants. Immunohistochemistry demonstrated intense staining of established fibrotic areas of the lung and expression related to the severity of lung function abnormalities. We confirmed increased expression of CD248 in IPF derived fibroblasts compared to normal primary pulmonary fibroblasts.

Formation of fibroblastic foci is a key feature reflecting sites of active on-going fibrogenesis. Increased numbers of fibroblastic foci have been associated with disease activity and a more rapid disease progression in IPF patients [2, 10]. Fibroblastic foci are often strong sources of TGF-β1 but expression is variable [26]. One unexpected finding in our study was that fibroblastic foci had less CD248 staining than the areas of established fibrosis. One explanation for this could be our finding that TGF-beta suppressed expression of CD248 in IPF derived fibroblasts in contrast to its effects on normal lung fiborblasts. (Fig. 3 and Additional file 1: Figure S4) A recent article by Babu et al. [27] suggests that the TGF-β1-mediated suppression of CD248 is present in normal murine embryonic fibroblasts but not murine lymphoma cell lines or in cancer-associated fibroblasts suggesting perhaps that the local cellular environment influences CD248 expression.

The fact, that TGF-β1 treatment leaves CD248 expression levels unchanged in normal lung fibroblasts but decreases the expression in IPF-derived cells (Fig. 3) allows us to speculate about the changes in the regulation of gene and protein expression occurring in IPF pathogenesis. Our results suggest that CD248 expression becomes newly regulated by TGF-beta signalling in IPF. Interestingly our experiments showed CD248 expression changes only on the protein expression but not on the mRNA levels (Fig. 3). This phenomenon is typical of post-transcriptional protein regulation, so we hypothesize that TGF-beta regulates CD248 protein levels by influencing the post-transcriptional mechanisms rather than the on the level of gene transcription. However, the identification of the exact regulatory mechanism is beyond the scope of this project. Higher CD248 expression has been associated in tumour-associated fibroblasts and pericytes while in normal stroma CD248 is hardly detectable [28]. In pulmonary fibrosis we observed elevated CD248 expression. Similarly Smith et al. described elevated CD248 expression in kidney fibrosis [21]. Since it is known that CD248 binds to ECM components, it is interesting to speculate that profound changes can be observed in ECM modelling/remodelling in both fibrotic and malignant diseases and how these changes might regulate CD248 expression levels and ECM-derived cues for cellular functions, like proliferation, adhesion and migration.

We found that CD248 is not expressed on pulmonary epithelial cells during TGF-β1-induced epithelial-mesenchymal transition, nor on a lung carcinoma cell line (Fig. 5). This is in concordance with the findings of Rouleau et al. [29] The authors describe high levels of CD248 expression in the stromal components but not on the malignant cells of carcinomas. Interestingly, the malignant cells of sarcomas proved to be highly positive for CD248 expression [19, 29]. These findings strongly suggest that CD248 is a genuine mesenchymal marker which is not induced by epithelial-mesenchymal transition in contrast to other mesenchymal proteins like S100A4 or vimentin [7, 30, 31]. Presently there is an ongoing debate on whether mesenchymal elements in fibrotic organs derive in large numbers from epithelial cells [7]. Although CD248 is not a fibroblast-specific marker [14, 24] these findings might highlight the usefulness of CD248 as a marker for separating mesenchymal-like cells originating from epithelial cells from genuine mesenchymal cells. This could aid appropriate flow sorting protocols in studies looking at epithelial mesenchymal interactions.

This research has its limitations. Firstly, immunohistochemistry is not a truly quantitative technique as DAB staining intensity shows no linear correlation with antigen expression levels [22]. That is why we chose to measure CD248 staining area instead of staining intensity. By using the area of extent of CD248 staining compared to the area stained by non-immune nuclear stain haematoxylin we did show significant correlations with disease severity and lung function. Further we demonstrated that IPF derived fibroblasts do express greater levels of CD248 than normal lung fibroblasts.

Secondly, whilst we demonstrated an inhibitory effect of CD248 siRNA upon proliferation, this was a relatively small effect but that may have been due to only 40-50 % efficiency of CD248 knockdown. We were unable to test the CD248 siRNA in IPF derived fibroblasts due to their limited availability. Finally, CD248 siRNA did not affect myofibroblast differentiation or collagen expression so the exact role CD248 plays in fibrogenesis is still unclear.

### Conclusion

In conclusion, CD248 appears to be a specific marker of mesenchymal cells that is elevated in the lungs of patients with IPF. It is noteworthy, that epithelial cells do not express CD248 when undergoing EMT, in contrast to other commonly used EMT biomarkers. CD248 expression correlated with markers of disease severity. CD248 was functionally important in terms of proliferation of primary pulmonary fibroblasts but does not appear to alter myofibroblast differentiation. CD248 may represent a novel therapeutic target in IPF to reduce fibro-proliferation.

### Ethical statement

All procedures in this study were performed in accordance with approval from the local research ethics committees at the University of Birmingham and Newcastle University. All patients included in this study gave written informed consent for the use of their tissue and clinical data for research purposes. Ethics committee approval number 07/MRE08/42.

### Consent for publication

Not applicable for this research.

### Availability of data and materials

All relevant data and materials are published in the manuscript and supplementary materials.

## Additional file

Additional file 1: Supplementary material. (PDF 523 kb)

## Abbreviations

a-SMA: alpha smooth muscle actin
ANOVA: analysis of variance
BrdU: 5-bromo-2′-deoxyuridine
CD: cluster of differentiation
DAB: diaminobenzidine
DMEM: Dulbecco’s modified Eagle’s Medium
DNA: deoxyribonucleic acid
Dnase: deoxyribonuclease
ECM: extracellular matrix
ELISA: enzyme-linked immunosorbent assay
EMT: epithelial-mesenchymal transition
FCS: fetal calf serum
FEV1: forced expiratory volume in 1 s
FITC: fluorescein-isothiocianate
FVC: forced vital capacity
HEPES: 4-(2-hydroxyethyl)-1-piperazineethanesulfonic acid
IHC: immunohistochemistry
ILD: interstitial lung diseases
IPF: idiopathic pulmonary fibrosis
KD: knock-down
MFI: median fluorescent intensitíy
NHLF: Normal Human Lung Fibroblasts
NHS: National Health Service
PCR: polimerase chain reaction
PDGF: platelet-derived growth factor
qPCR: quantitative polimerase chain reaction
r2: r squared
RNA: ribonucleic acid
siRNA: silencing ribonucleic acid
TEM-1: tumour endothelial marker-1
TGF-β1: transforming growth factor beta 1
TLC: total lung capacity
TLCO: transfer factor for carbon monoxide
VATS: video-assisted thoracoscopy
