# Supplementary material for: Role of CD248 as a potential severity marker in idiopathic pulmonary fibrosis
Source: BMC Pulm Med. 2016 Apr 14;16:51. doi: 10.1186/s12890-016-0211-7 (PMC4832513; doi:10.1186/s12890-016-0211-7)
Supplement: Supplementary file 1 — Supplementary material. (PDF 523 kb) [file 12890_2016_211_MOESM1_ESM.pdf]

Supplementary material

Role of CD248 as a potential severity marker in idiopathic pulmonary fibrosis

Domokos Bartis, Louise E Crowley, Vijay K D’Souza, Lee Borthwick, Andrew J Fisher,
Adam P Croft, Judit E Pongracz, Richard Thompson, Gerald Langman,
Cristopher D Buckley, David R Thickett

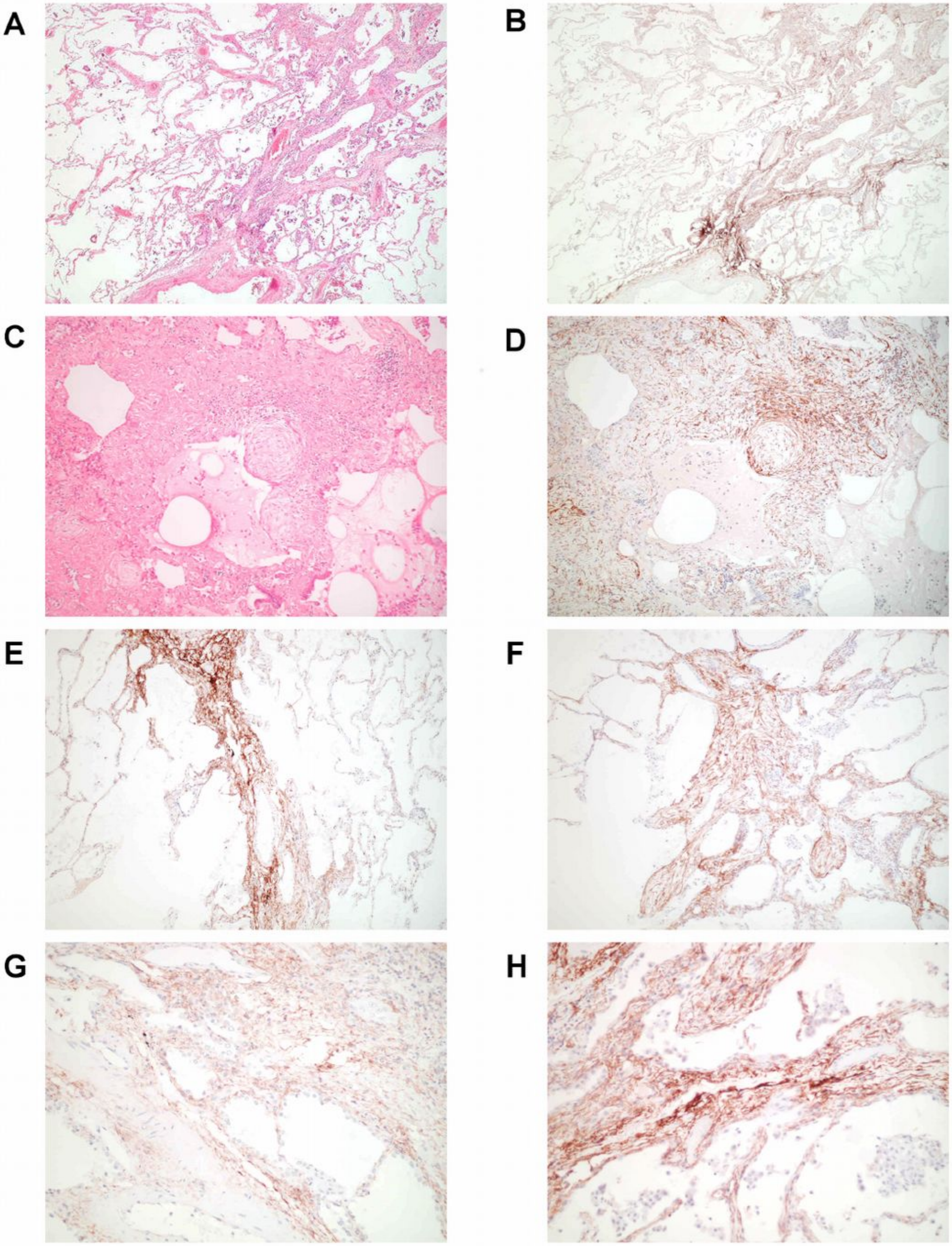

Supplementary figure 1. CD248 IHC showing staining of various lung structures.

A: H&E staining reveals a classic case of UIP showing moderate fibrosis alternating with normal lung tissue. Inflammation is minimal (magnification: 40x)
B: CD248 staining (brown) of the corresponding area as shown in (A), showing stron CD248 staining in fibrotic areas but no staining in uninvolved lung tissue (magnification 40x)
C: H&E stain showing fibroblastic foci (magnification 100x).
D: Same area depicted as in (C). CD248 staining (brown) is moderate or absent fibroblastic foci, but remarkably stronger in surrounding fibrotic areas.
E: Very strong CD248 staining i the interlobular fissures (magnification 200x)
F: strong CD248 staining in peribronchial fibrosis (magnification 100x)
G: Mild-to-moderate CD248 staining in the fibrotic interstitium (magnification 200x)
H: Strong CD248 staining in fibrotic areas but not in uninvolved lung tissue (magnification 200x)

Supplementary Figure 2

A

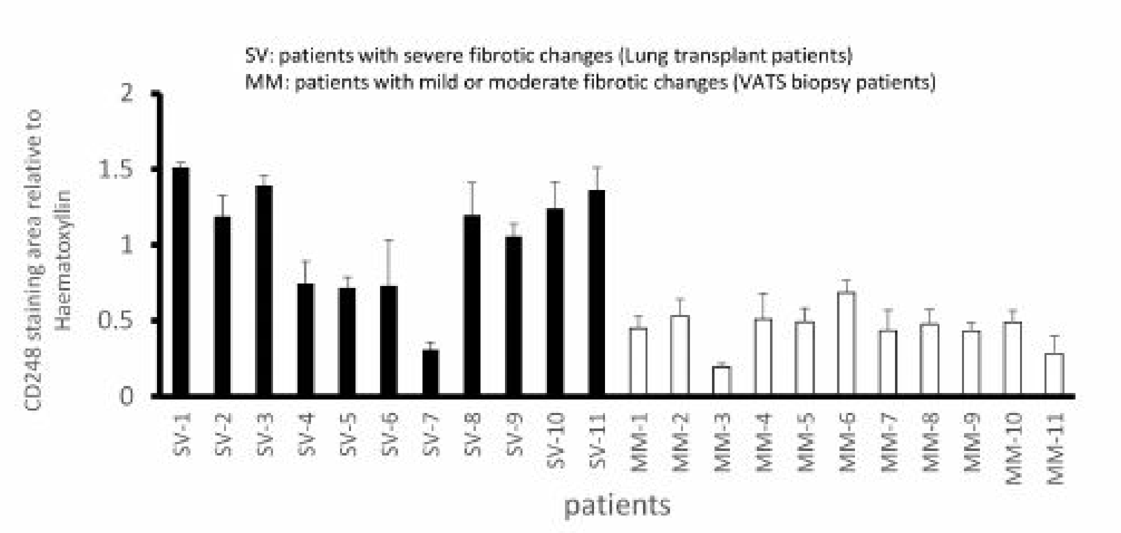

B

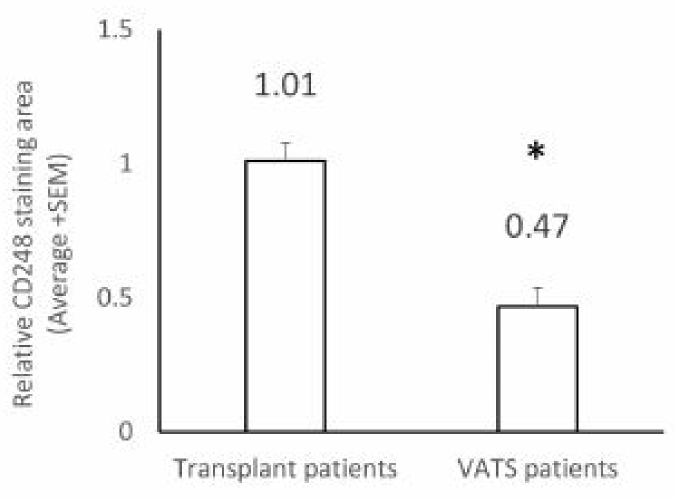

Supplementary figure 2 Panel A.
Relative CD248 staining area of tissue samples of individual patients is shown, bars represent average ± SEM. Closed bars show IPF patients with severe fibrotic changes (explanted lungs of lung transplanted patients, n=11, marked SV1-SV11) and open bars show VATS biopsy patients with mild-to-moderate fibrotic changes, (n=11, marked MM1-MM11). We took 3-7 high resolution digital images of each section at random locations. For deteiled analysis methods please see the manuscript methods section.
Supplementary figure 2 Panel B.
On the diagram, the average ± SEM of relative CD248 staining area of transplant patients (n=11) and VATS patients is shown. We used both Student`s t-test (* $p<10^{-8}$) and Mann-Whitney U-test (* $p<10^{-12}$) for statistical analysis of the data derived from images.

Supplementary Figure 3

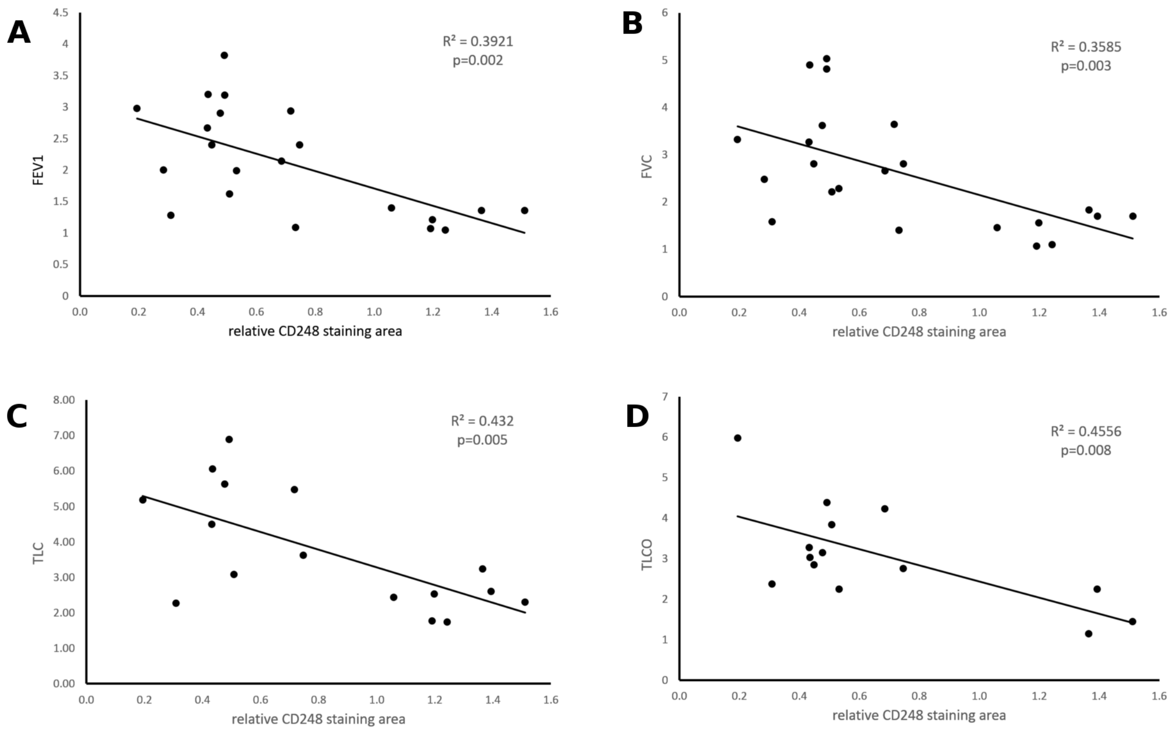

Supplementary figure 3 Panel A: Forced Expiratory Volume in 1 second (FEV1) values show a significant (ANOVA, $p<0.01$) negative correlation in IPF patients when compared to relative CD248 staining area. Dots represents individual IPF patients with mild-to-moderate (n=11) and severe fibrosis (n=11), R^2 value (0.3921) and regression line fitting was calculated using linear regression. **Panel B:** Forced Vital Capacity (FVC) values show a significant (ANOVA, $p<0.01$) negative correlation in IPF patients when compared to relative CD248 staining area. Dots represents individual IPF patients with mild-to-moderate (n=11) and severe fibrosis (n=11). R^2 value (0.3585) and regression line fitting was calculated using linear regression. **Panel C:** Total Lung Capacity (TLC) values show a significant (ANOVA, $p<0.01$) negative correlation in IPF patients when compared to relative CD248 staining area. Dots represents individual IPF patients with mild-to-moderate (n=11) and severe fibrosis (n=11), R^2 value (0.4320) and regression line fitting was calculated using linear regression. **Panel D:** The transfer factor for carbon monoxide (T_{LCO})values show a significant (ANOVA, $p<0.01$) negative correlation in IPF patients when compared to relative CD248 staining area. Dots represents individual IPF patients with mild-to-moderate (n=11) and severe fibrosis (n=11), R^2 value (0.4556) and regression line fitting was calculated using linear regression.

Supplementary Figure 4.

A

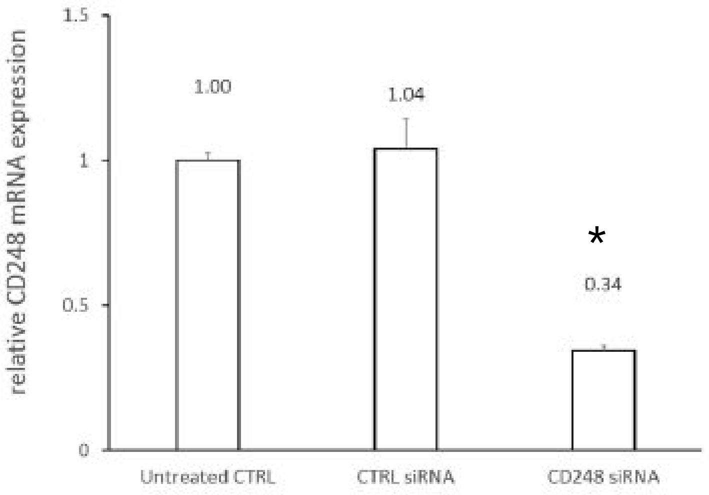

B

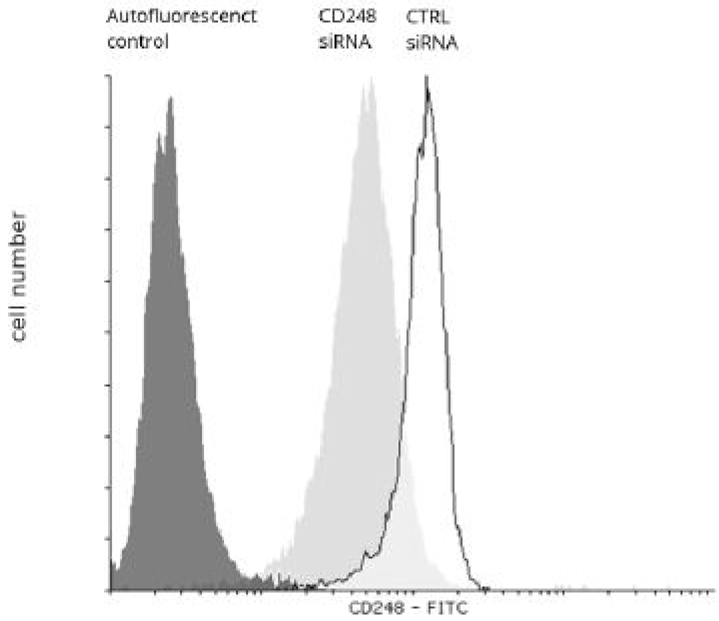

Supplementary figure 4. Panel A.
CD248 mRNA expression levels is shown relative to GAPDH. NHLFs were transfected with scrambled or CD248-specific siRNAs or left untreated, as described in the methods section. CD248 expression levels did not significantly differ from that of when NHLF were transfected with scrambled siRNA control but sequence-specific CD248 siRNA significantly (*$p<0.05$) lowered CD248 mRNA levels compared to both untreated or scrabled siRNA-transfected cells.

Supplementary figure 4. Panel B.
CD248 protein expression levels were measured using flow cytometry. NHLF siRNA transfection and fluorescent labelling was performed as described in the methods section. Graph represent a typical CD248 KD result. Open histogram represents CD248 protein expression levels on NHLF cells trasfected with scramled siRNA. NHLFs show lower CD248 expression when transfected with CD248-specific siRNA (grey closed histogram). Black closed histogram show the autofluorescence of untransfected siRNA.

Supplementary table 1. List of primers used in experiments

Source	Target	Assay ID	Catalogue Number
**TaqMan® Gene Expression Assay (Life Technologies)**	CD248	Hs00535586_s1	4331182
	Glyceraldehyde 3-phosphate dehydrogenase (GAPDH)	AI1Q8N4	4331182
	Collagen , type 1, α1 (COL1A1)	Hs00164004_m1	4331182
	**Target**	**Forward Sequence (left)**	**Reverse Sequence (right)**
**Primers (from Alta Bioscience Birmigham, UK)**	Homo sapiens actin, alpha 2, smooth muscle	CCGACCGAATGCAGAAGGA	ACAGAGTATTTGCGCTCCGAA
	human Vimentin	CTTCAGAGAGAGGAAGCCGA	ATTCCACTTTGCGTTCAAGG
	human SNAIL homolog 2 (SLUG)	CAGACCCTGGTTGCTTCAA	TGACCTGTCTGCAAATGCTC
